# Assessment of H3K27me3 immunohistochemistry and combination of *NF1* and *p16* deletions by fluorescence *in situ* hybridization in the differential diagnosis of malignant peripheral nerve sheath tumor and its histological mimics

**DOI:** 10.1186/s13000-021-01140-0

**Published:** 2021-08-30

**Authors:** Shintaro Sugita, Tomoyuki Aoyama, Makoto Emori, Tomomi Kido, Tomoko Takenami, Kodai Sakuraba, Kotomi Terai, Taro Sugawara, Mitsuhiro Tsujiwaki, Tadashi Hasegawa

**Affiliations:** 1 Department of Surgical Pathology, Sapporo Medical University, School of Medicine, South 1, West 16, Chuo-ku, Sapporo, Hokkaido 060-8543 Japan; 2 Department of Orthopedic Surgery, Sapporo Medical University, School of Medicine, Sapporo, Hokkaido 060-8543 Japan

**Keywords:** Malignant peripheral nerve sheath tumor, *NF1* deletion, *p16* deletion, Fluorescence *in situ* hybridization, H3K27me3, Immunohistochemistry

## Abstract

**Background:**

A definitive diagnosis of malignant peripheral nerve sheath tumor (MPNST) is challenging, especially in cases without neurofibromatosis 1 (NF1), because MPNST lacks specific markers on immunohistochemistry (IHC).

**Methods:**

We performed IHC for histone 3 trimethylated on lysine 27 (H3K27me3) and evaluated the percentage of cells with H3K27me3 loss using measured values at 10% intervals, categorized as complete loss (100% of tumor cells lost staining), partial loss (10% to 90% of tumor cells lost staining), and intact (no tumor cells lost staining). We conducted fluorescence *in situ* hybridization (FISH) for *NF1* and *p16* deletions comparing 55 MPNSTs and 35 non-MPNSTs, consisting of 9 synovial sarcomas (SSs), 8 leiomyosarcomas (LMSs), 10 myxofibrosarcomas (MFSs), and 8 undifferentiated pleomorphic sarcomas (UPSs). We assessed the percentage of cells with homozygous and heterozygous deletions and defined “deletion” if the percentage of either the *NF1* or *p16* deletion signals was greater than 50% of tumor cells.

**Results:**

Among the 55 MPNSTs, 23 (42%) showed complete H3K27me3 loss and 32 (58%) exhibited partial loss or intact. One each of the 9 SSs (11%), 8 LMSs (12%), and 8 UPSs (12%) showed complete H3K27me3 loss and many non-MPNSTs exhibited intact or partial H3K27me3 loss. Among the 55 MPNSTs, 33 (60%) and 44 (80%) showed *NF1* or *p16* deletion, respectively. Co-deletion of *NF1* and *p16* was observed in 29 (53%) MPNSTs. Among the 23 MPNTSs showing H3K27me3 complete loss, 18 (78%) and 20 (87%) exhibited *NF1* or *p16* deletion, respectively. Among the 32 MPNSTs with H3K27me3 partial loss or intact, 15 (47%) and 24 (75%) exhibited *NF1* or *p16* deletion, respectively. The frequency of *NF1* and/or *p16* deletion tended to be lower in non-MPNSTs than in MPNSTs. Approximately 90% of MPNSTs included cases with H3K27me3 complete loss and cases showing H3K27me3 partial loss or intact with *NF1* and/or *p16* deletion. Approximately 50% of MPNSTs showed co-deletion of *NF1* and *p16* regardless of H3K27me3 loss.

**Conclusions:**

FISH for *NF1* and *p16* deletions, frequently observed in high-grade MPNSTs, might be a useful ancillary diagnostic tool for differentiating MPNST from other mimicking spindle cell and pleomorphic sarcomas.

## Background

Malignant peripheral nerve sheath tumor (MPNST) is characterized by differentiation toward peripheral nerve sheath tissue. Approximately 50% of MPNST cases are associated with neurofibromatosis 1 (NF1), which is the most important clinical parameter for a definitive diagnosis of MPNST. Diagnosis of MPNST without NF1 is sometimes challenging because there are currently no established markers for MPNST on immunohistochemistry (IHC). Classically, MPNSTs express S-100 protein sparsely, but clearly, on IHC. However, the intensity of S-100 protein expression is sometimes markedly diminished and non-specific S-100 protein expression is often observed in other sarcomas. Recent studies have revealed that histone 3 trimethylated on lysine 27 (H3K27me3) is a useful diagnostic marker for MPNST [1, 2]. MPNST often shows complete or so-called “mosaic” loss of H3K27me3 expression on IHC, and loss of H3K27me3 is the basis for diagnosing MPNST. However, approximately one-third of cases retain H3K27me3 expression, and the evaluation of H3K27me3, especially the detailed ratio of mosaic loss, is empirically difficult and seems to be somewhat non-objective. In the clinical setting, we make a final diagnosis of MPNST by the combination of H3K27me3 expression and classic diagnostic hallmarks including S-100 protein expression and association with NF1.

It has been reported that *NF1* deletion is characteristic of MPNST. *NF1* deletion can be detected by fluorescence *in situ* hybridization (FISH) and is useful in pathological diagnosis using formalin-fixed and paraffin-embedded specimens. Perry et al. found that *NF1* was characteristically deleted in MPNST cases in a study of benign peripheral nerve sheath tumors and MPNSTs [3]. In addition, Suzuki et al. showed that *NF1* deletion FISH was useful for diagnosis in diagnostically challenging cases of intraosseous MPNST without NF1 [4].

Perry et al. first revealed the diagnostic utility of a FISH assay for *NF1* and *p16* in MPNST [5]. They performed a dual-color FISH assay with 22 MPNSTs and benign and malignant non-peripheral nerve sheath tumors (non-MPNSTs) including 13 plexiform schwannomas, 5 cellular schwannomas, 8 synovial sarcomas, 6 fibrosarcomas, and 13 hemangiopericytomas. They demonstrated the specific deletion of *NF*1 and *p16* in MPNST and indicated that homozygous and heterozygous deletion of *p16* might distinguish MPNST from benign nerve sheath tumor and other histologically mimicking sarcomas. From the genetic aspect, *p16/CDKN2A* inactivation is considered a key event in the occurrence and progression of MPNST. Loss of the *p16/CDKN2A* locus at 9p21 is one of the earliest events in the malignant transformation of neurofibromas. The expression of p16 on IHC is sometimes decreased in MPNST [6]. Atypical and conventional neurofibromas also exhibit the loss of nuclear p16 expression. Therefore, *p16/CDKN2A* inactivation may be an early change in malignant progression [7].

In this study, aiming for more accurate differential diagnosis, we examined the diagnostic utility of FISH for *NF1* and *p16* deletions in the differential diagnosis of MPNST and its mimicking spindle cell and pleomorphic sarcomas, especially in mosaic loss cases in which the interpretation of H3K27me3 can be difficult.

## Methods

### Sample selection

This study was performed with the approval of the Institutional Review Board (IRB) of Sapporo Medical University Hospital (No. 272-107). The IRB approved an opt-out informed consent approach for a retrospective, non-interventional study. We selected 55 MPNST cases from our pathological archives. Twenty cases were NF1-associated MPNSTs (NF1Ms) and 35 cases were non-NF1-associated MPNSTs (NNF1Ms). We determined histological grade using the parameters of tumor differentiation, mitotic figures, and tumor necrosis according to the Fédération Nationale des Centres de Lutte Contre le Cancer grading system. We performed hematoxylin and eosin staining using 3-μm-thick sections. We reviewed all hematoxylin and eosin-stained slides and previously stained IHC slides in individual cases. Next, we selected 35 non-MPNST sarcomas that needed to be distinguished from MPNST, comprising 9 synovial sarcomas (SSs), 8 leiomyosarcomas (LMSs), 10 myxofibrosarcomas (MFSs), and 8 undifferentiated pleomorphic sarcomas (UPSs). The histopathological criteria for non-MPNSTs were as follows. SS showed fascicular proliferation of uniform spindle cells. The tumor cells were positive for cytokeratin and/or epithelial membrane antigen to varying degrees on IHC. All SS cases had been shown to have *SS18* rearrangement by FISH. LMS exhibited interlacing fascicules of spindle cells with cigar-shaped nuclei and eosinophilic cytoplasm. The tumor cells expressed at least two of three myogenic markers (α-smooth muscle actin, desmin, and muscle-specific actin) on IHC. MFS was characterized by multinodular growth of hypocellular proliferating atypical spindle cells with prominent elongated, curvilinear, thin-walled blood vessels in a myxoid background. UPS exhibited a pattern-less growth of severely atypical spindle or pleomorphic cells without any differentiation toward specific tissue on IHC.

We checked the immunoreactivity of previously performed IHC for S-100 protein in all 55 MPNST cases. As a result, 50 cases (approximately 90%) expressed S-100 protein on IHC. Twenty-eight cases demonstrated sparse or focal expression of S-100 protein. On the other hand, 22 cases were diffusely positive for S-100 protein.

### H3K27me3 IHC

We performed IHC for H3K27me3 using representative sections from formalin-fixed paraffin-embedded tissues from MPNST and non-MPNST cases. These tissues were sliced into 3-μm-thick sections and examined with an automated IHC system at Sapporo Medical University Hospital. All slides were loaded into a PT Link Module (Agilent Technologies, Santa Clara, CA) and subjected to a heat-induced antigen-retrieval protocol with EnVision FLEX Target Retrieval Solution (Agilent Technologies) before being transferred to an Autostainer Link 48 (Agilent Technologies). We used antibodies against H3K27me3 (C36B11, 1:200 dilution; Cell Signaling Technology, Danvers, MA). We determined H3K27me3 loss when we recognized the loss of H3K27me3 nuclear staining in the tumor cells. We evaluated the percentage of cells with H3K27me3 loss using measured values at 10% intervals and categorized it as complete loss (100% of tumor cells lost staining), partial loss (10% to 90% of tumor cells lost staining), and intact (no tumor cells lost staining).

### FISH

We performed FISH using the commercially available probes MD NF1 (17q11)/MPO (17q22) (Leica, Wetzlar, Germany) for *NF1* deletion and Vysis LSI CDKN2A SpectrumOrange/CEP9 SpectrumGreen Probes (Abbott, Abbott Park, IL) for *p16* (CDKN2A) deletion. Each of the probes was labeled with red or green dye for the target or control locus, respectively. FISH was performed as described previously [8]. In brief, the specimens were tumor tissues in 4-μm-thick slices on glass slides. We first selected an area showing representative histology and marked a 5-mm circle with a marker pen on the glass slide. We used a PathVysion HER-2 DNA Probe Kit (Abbott) and followed the manufacturer’s procedure, with modifications: baking (60°C for 1 h), deparaffinization, target gene activation (20 min with 0.2 M HCl followed by 80°C for 30 min with pretreatment solution), enzyme treatment (37°C for 60 min with protease solution), re-fixation (10 min with 10% formalin neutral buffer solution), denaturation (72°C for 5 min with denaturation solution), washing and dehydration (1 min each in 70%, 85%, and 100% ethanol), hybridization with 10 mL DNA probe solution (90°C for 5 min, followed by 37°C for 48 h), and washing with post-hybridization wash buffer (72°C for 2 min). For counterstaining, 10 μL of 4,6-diamidino-2-phenylindole was added. The slides were cover-slipped for viewing under a fluorescence microscope. We counted 50 tumor nuclei and calculated the percentage of cells with deletion signals. Deletion signals were categorized as homozygous deletion, heterozygous deletion, and monosomy. Homozygous deletion was defined as complete deletion of both alleles of the target locus and cells showed only control green signals. Heterozygous deletion was defined as alternate deletion of one of the alleles of the target locus, and so the number of red signals for the target locus was less than the number of green signals for the control locus. Tumor cells with monosomy had one allele, which showed a pair of red and green signals. Monosomy was considered a variation of heterozygous deletion. We defined “deletion” if the percentage of either the *NF1* or *p16* deletion signals was greater than 50% of tumor cells.

### Statistical analysis

To examine whether there was a difference in the IHC and FISH results between low-grade and high-grade MPNST, we performed statistical analysis by Fisher's exact test using IBM SPSS Statistics 25 software (IBM SPSS Statistics, Chicago, IL). For all analysis, differences at P < 0.05 were considered statistically significant.

## Results

### Histological examinations

**Fig. 1:**
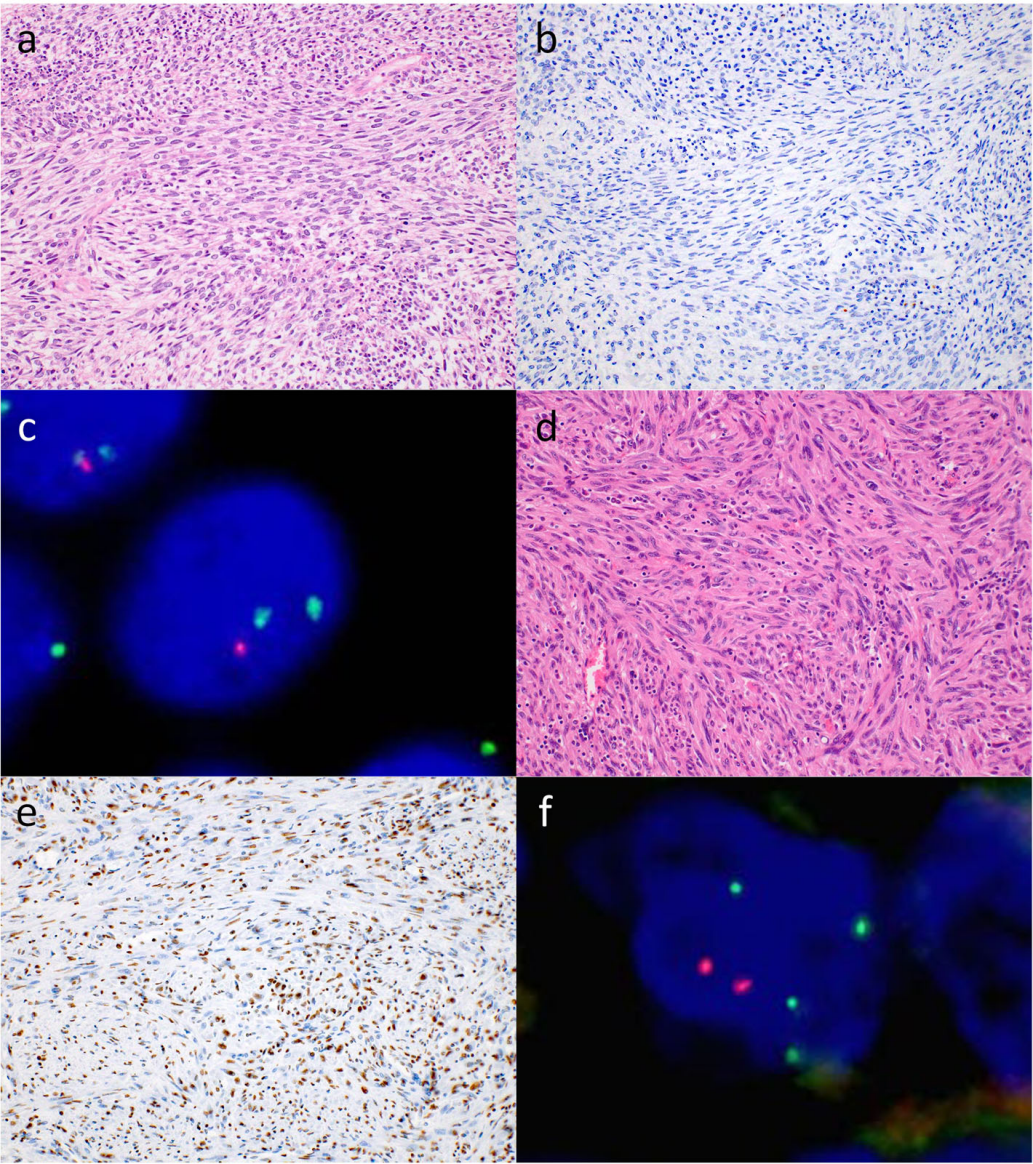
Pathological findings including morphology, immunohistochemistry (IHC), and fluorescence *in situ* hybridization (FISH) of malignant peripheral nerve sheath tumor (MPNST) cases. **a**. MPNST with fascicular proliferation of spindle cells that had enlarged oval to spindle nuclei with moderate to severe nuclear atypia (NF1M-20). **b**. IHC for histone 3 trimethylated on lysine 27 (H3K27me3). The tumor cells were completely negative for H3K27me3 on IHC (NF1M-20). **c**. FISH for *p16* deletion. The tumor nuclei showed heterozygous deletion of *p16*. Two green signals indicating a control locus and one red signal indicating a target locus were found (NF1M-20). **d**. Another case of MPNST also exhibited fascicular and storiform proliferation of spindle cells with enlarged spindle nuclei with moderate to severe nuclear atypia (NF1M-6). **e**. IHC for H3K27me3. A majority of tumor cells were positive for H3K27me3 on IHC, although H3K27me3-negative tumor cells were focally intermingled. The tumor showed partial loss of H3K27me3 (NF1M-6). **f**. FISH for neurofibromatosis 1 (*NF1*) deletion. Tumor nuclei showing heterozygous deletion of *NF1*. The number of red signals (two) for the target locus was less than the number of green signals (four) for the control locus (NF1M-6)

Histologically, MPNST consisted mainly of fascicular and storiform proliferation of spindle cells that had enlarged oval to spindle nuclei with moderate to severe nuclear atypia and nuclear pleomorphism (Fig. 1a, d). The tumor showed a marble pattern intermingled with solid and edematous to myxoid areas. Mitotic figures were frequently observed with necrotic foci. Some MPNSTs consisted of the solid proliferation of round cells. Some cases also showed a myxoid morphology, and glandular differentiation foci and scattered rhabdomyoblasts (malignant Triton tumor) were observed. Our cohort consisted of 7 cases of low-grade/grade 1 MPNST and 48 cases of high-grade/grades 2 and 3 MPNST.

### H3K27me3 IHC

**Fig. 2:**
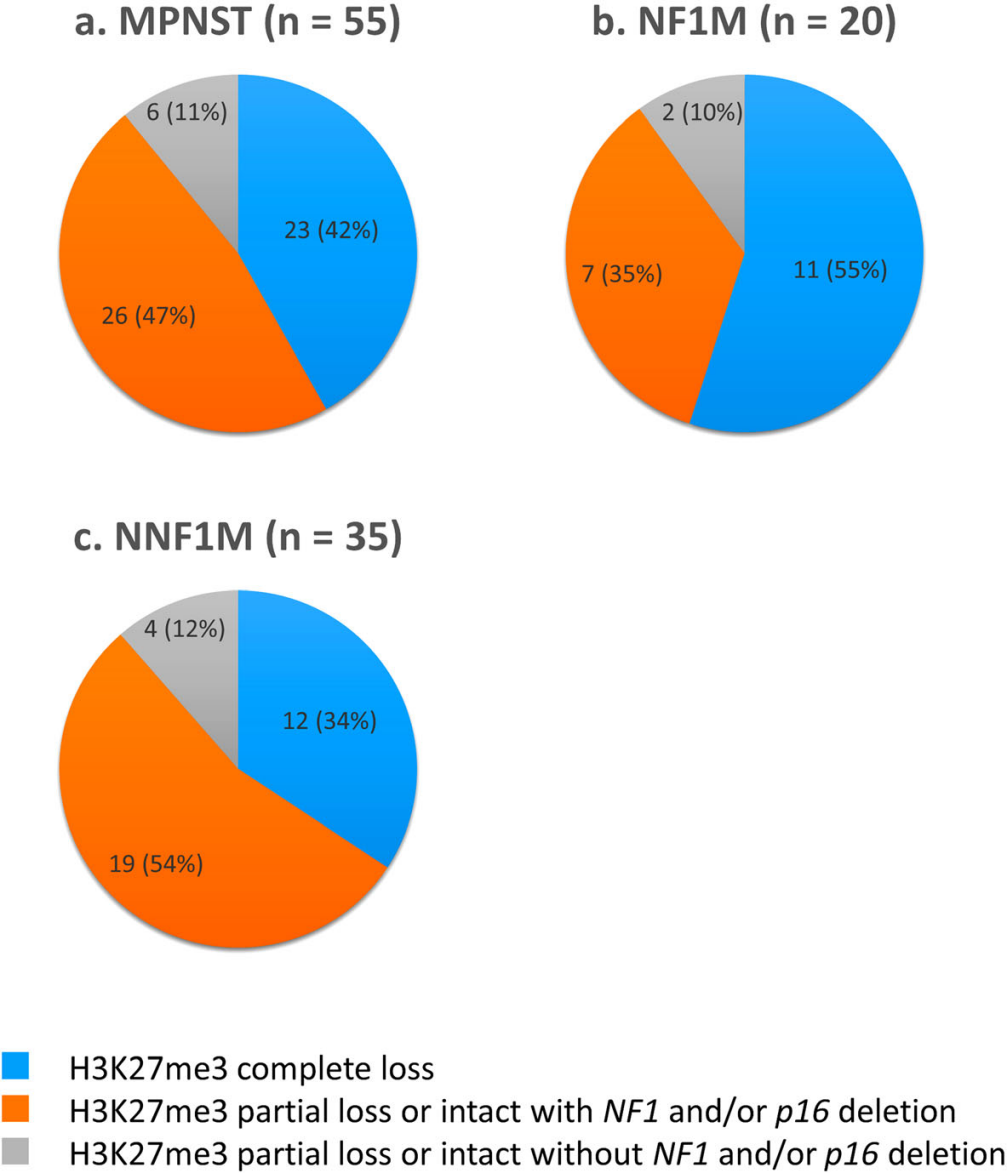
Combination of immunohistochemistry for histone 3 trimethylated on lysine 27 (H3K27me3) and fluorescence *in situ* hybridization for neurofibromatosis 1 (*NF1*) and *p16* deletion in malignant peripheral nerve sheath tumor (MPNST) cases. **a**. Status of H3K27me3 loss and *NF1* and/or *p16* deletion in all MPNST cases. A majority of MPNST cases had H3K27me3 complete loss and H3K27me3 partial loss or intact with *NF1* and/or *p16* deletion. **b**. Status of H3K27me3 loss and *NF1* and/or *p16* deletion in neurofibromatosis 1 (NF1)-associated MPNST (NF1M) cases. **c**. Status of H3K27me3 loss and *NF1* and/or *p16* deletion in non-NF1 associated MPNST (NNF1M) cases

**Fig. 3:**
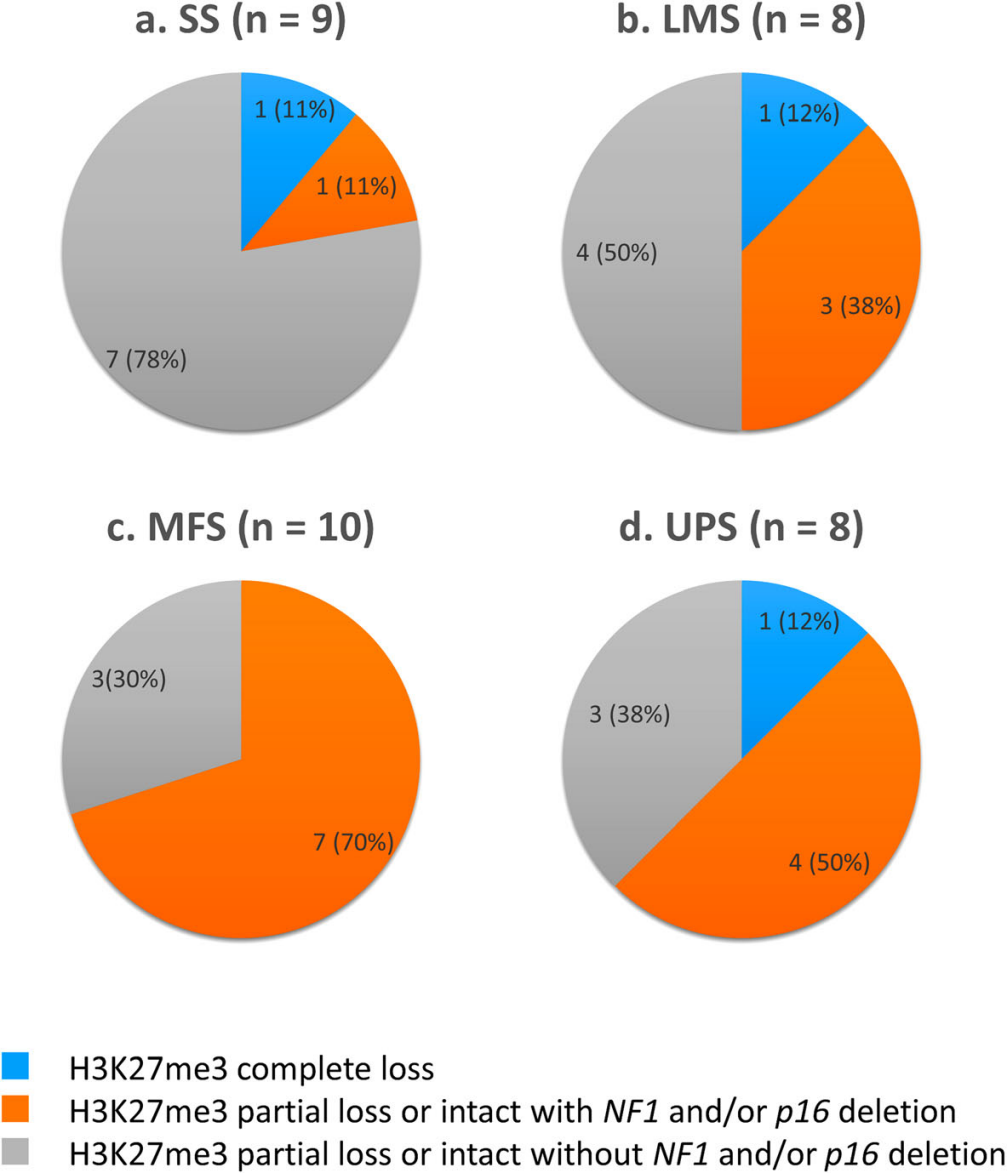
Combination of immunohistochemistry for histone 3 trimethylated on lysine 27 (H3K27me3) and fluorescence *in situ* hybridization for neurofibromatosis 1 (*NF1*) and *p16* deletion in non-malignant peripheral nerve sheath tumors. **a**. Status of H3K27me3 loss and *NF1* and/or *p16* deletion in synovial sarcoma (SS) cases. A minority of SS cases showed H3K27me3 complete loss and H3K27me3 partial loss or intact with *NF1* and/or *p16* deletion. **b**. Status of H3K27me3 loss and *NF1* and/or *p16* deletion in leiomyosarcoma (LMS) cases. **c**. Status of H3K27me3 loss and *NF1* and/or *p16* deletion in myxofibrosarcoma (MFS) cases. A majority of MFS cases showed H3K27me3 partial loss or intact with *NF1* and/or *p16* deletion. **d**. Status of H3K27me3 loss and *NF1* and/or *p16* deletion in undifferentiated pleomorphic sarcoma (UPS) cases

We performed H3K27me3 IHC in 55 cases of MPNST (20 NF1Ms and 35 NNF1Ms) and 35 non-MPNST cases (Table 1). Among the 55 MPNSTs, 23 (42%) showed complete loss of H3K27me3 on IHC (Figs. 1b, 2a) and 32 (58%) exhibited partial loss or intact (Figs. 1e, 2a). Among the 20 NF1Ms and 35 NNF1Ms, 11 (55%) (Fig. 2b) and 12 (34%) (Fig. 2c) showed complete loss of H3K27me3, respectively. There was no difference in the staining pattern between the conventional MPNST cases and those with heterologous components (malignant Triton tumor). On the other hand, one each of the 9 SSs (11%), 8 LMSs (12%), and 8 UPSs (12%) showed complete loss of H3K27me3, and many non-MPNSTs exhibited intact or partial loss of H3K27me3 (Fig. 3).

**Table 1:** Status of H3K27me3 loss, *NF1* deletion, and *p16* deletion in individual cases of MPNST and non-MPNST

Case	H3K27me3 loss (%)	*NF1* del (%)	*p16* del (%)	Case	H3K27me3 loss (%)	*NF1* del (%)	*p16* del (%)	Case	H3K27me3 loss (%)	*NF1* del (%)	*p16* del (%)
NNF1M-1	0	0	0	NF1M-1	0	6	48	SS-1	10	24	28
NNF1M-2	0	20	32	NF1M-2	0	48	94	SS-2	10	32	20
NNF1M-3	0	47	100	NF1M-3	10	12	40	SS-3	10	70	40
NNF1M-4	0	20	58	NF1M-4	10	96	96	SS-4	40	8	20
NNF1M-5	0	20	56	NF1M-5	10	84	88	SS-5	50	18	16
NNF1M-6	0	38	76	NF1M-6	10	72	98	SS-6	60	6	32
NNF1M-7	0	38	100	NF1M-7	20	6	68	SS-7	70	10	16
NNF1M-8	0	88	56	NF1M-8	70	90	30	SS-8	70	22	14
NNF1M-9	10	0	76	NF1M-9	90	98	14	SS-9	100	50	42
NNF1M-10	10	12	14	NF1M-10	100	38	66	LMS-1	10	84	96
NNF1M-11	10	10	70	NF1M-11	100	20	56	LMS-2	20	12	10
NNF1M-12	10	12	90	NF1M-12	100	96	14	LMS-3	20	38	98
NNF1M-13	10	24	74	NF1M-13	100	84	84	LMS-4	30	26	28
NNF1M-14	10	68	66	NF1M-14	100	60	94	LMS-5	40	14	90
NNF1M-15	10	80	82	NF1M-15	100	62	62	LMS-6	70	18	18
NNF1M-16	10	60	72	NF1M-16	100	60	80	LMS-7	90	14	36
NNF1M-17	10	88	78	NF1M-17	100	76	88	LMS-8	100	12	22
NNF1M-18	10	62	88	NF1M-18	100	96	98	MFS-1	10	24	90
NNF1M-19	20	44	12	NF1M-19	100	78	58	MFS-2	20	8	84
NNF1M-20	30	100	64	NF1M-20	100	96	98	MFS-3	30	48	16
NNF1M-21	30	98	56					MFS-4	30	4	96
NNF1M-22	70	72	98					MFS-5	40	84	80
NNF1M-23	80	56	68					MFS-6	80	10	30
NNF1M-24	100	10	8					MFS-7	90	22	48
NNF1M-25	100	36	92					MFS-8	90	46	80
NNF1M-26	100	20	60					MFS-9	90	48	100
NNF1M-27	100	78	36					MFS-10	90	54	98
NNF1M-28	100	88	98					UPS-1	0	30	40
NNF1M-29	100	60	94					UPS-2	0	66	96
NNF1M-30	100	66	82					UPS-3	10	46	44
NNF1M-31	100	58	88					UPS-4	10	40	14
NNF1M-32	100	64	80					UPS-5	10	76	74
NNF1M-33	100	86	94					UPS-6	10	52	94
NNF1M-34	100	96	84					UPS-7	80	6	52
NNF1M-35	100	52	70					UPS-8	100	40	44

*del* deletion, *H3K27me3* histone 3 trimethylated on lysine 27, *LMS* leiomyosarcoma, *MFS* myxofibrosarcoma, *MPNST* malignant peripheral nerve sheath tumor, *NF1* neurofibromatosis 1, *NF1M* neurofibromatosis 1-associated malignant peripheral nerve sheath tumor, *NNF1M* non-neurofibromatosis 1-associated malignant peripheral nerve sheath tumor, *SS* synovial sarcoma, *UPS* undifferentiated pleomorphic sarcoma

### FISH

The results for *NF1* and *p16* deletion FISH of all cases are described in Table 1. Among the 55 MPNSTs, 33 (60%) and 44 (80%) showed *NF1* or *p16* deletion, respectively. Co-deletion of *NF1* and *p16* was observed in 29 (53%) MPNSTs. Among the 23 MPNSTs showing H3K27me3 complete loss, 18 (78%) and 20 (87%) exhibited *NF1* or *p16* deletion, respectively. Among the 32 MPNSTs with H3K27me3 partial loss or intact, 15 (47%) showed *NF1* deletion and 24 (75%) exhibited *p16* deletion. On the other hand, although MFSs often showed *p16* deletion, the frequency of *NF1* and *p16* deletion tended to be lower in non-MPNSTs than in MPNSTs.

On fluorescence microscopy, FISH typically detected only two green signals, indicating homozygous deletion of *NF1* in MPNSTs. On the other hand, heterozygous deletion of *NF1* was indicated by the lower number of red signals for the target locus compared to the number of green signals for the control locus (Fig. 1f). FISH also detected *p16* homozygous deletion, showing only green signals. Additionally, *p16* heterozygous deletion was identified, in which the number of red signals for the target locus was less than the number of green signals for the control locus (Fig. 1c).

### Combination of H3K27me3 IHC and *NF1* and *p16* deletions by FISH for differential diagnosis

Among the 55 MPNSTs, 26 (47%) showed H3K27me3 partial loss or intact with *NF1* and/or *p16* deletion. Six cases (11%) exhibited H3K27me3 partial loss or intact without *NF1* and/or *p16* deletion. NF1Ms and NNF1Ms also demonstrated similar trends to the results of all MPNST cases (Fig. 2). Therefore, approximately 90% of MPNSTs included cases with H3K27me3 complete loss and cases showing H3K27me3 partial loss or intact with *NF1* and/or *p16* deletion. Approximately 50% of MPNSTs showed co-deletion of *NF1* and *p16*, regardless of the loss of H3K27me3 (Table 1 and Fig. 2). Among the non-MPNSTs, SSs included a minority of cases with H3K27me3 complete loss and cases showing H3K27me3 partial loss or intact with *NF1* and/or *p16* deletion. On the other hand, MFSs and UPSs mainly contained cases showing H3K27me3 partial loss or intact with/without *NF1* and/or *p16* deletion (Fig. 3).

### Statistical analysis

There was a significant difference in the presence of *NF1* and/or *p16* deletion between low-grade and high-grade MPNSTs (P < 0.022) (Table 2). There was no significant difference in the presence of H3K27me3 complete loss, *NF1* deletion, and *p16* deletion between both groups.

**Table 2:** Status of *NF1* and/or *p16* deletion between low-grade and high-grade MPNSTs

		*NF1* and/or *p16* deletion		
Histology	Total, *n* (%)	Present, *n* (%)	Absent, *n* (%)	P-value
Low-grade MPNST	7 (12.7)	4 (57.1)	3 (42.9)	0.022
High-grade MPNST	48 (87.3)	45 (93.8)	3 (6.2)	

*MPNST* malignant peripheral nerve sheath tumor, *NF1* neurofibromatosis 1

## Discussion

In general, a definitive diagnosis of MPNST is often difficult or challenging because the tumor fundamentally shows spindle cell morphology and does not possess any IHC markers that are completely specific for MPNST. The differential diagnosis of MPNST ranges from benign to malignant tumors [7], and so we investigated SS, LMS, MFS, and UPS. SS shows fascicular proliferation of spindle cells, especially in monophasic fibrous SS, and expresses epithelial markers (cytokeratin and epithelial membrane antigen) on IHC. Finally, SS has a specific chimeric *SS18-SSX* fusion gene. LMS also shows fascicular proliferation of spindle cells with cigar-like nuclei and eosinophilic cytoplasm. The tumor cells often express myogenic markers (α-smooth muscle actin, desmin, and muscle-specific actin) on IHC. MFS exhibits loose fascicular proliferation of atypical spindle cells with abundant myxoid matrix. UPS consists of markedly atypical spindle and pleomorphic cells. Neither MFS nor UPS have characteristic markers on IHC. Therefore, MPNST should always be differentiated from these tumors.

On IHC, MPNST often shows complete or mosaic loss of H3K27me3 expression. However, the specificity of the loss of H3K27me3 expression in the differential diagnosis of MPNST is not complete and one study reported that H3K27me3 cannot be used safely to differentiate between MPNST and malignant melanoma [9]. In addition, the loss of H3K27me3 expression has been confirmed in other histologic mesenchymal tumors including some extraskeletal osteosarcomas and dedifferentiated chondrosarcomas [10, 11].

A recent study revealed the diagnostic utility of histone H3K27 dimethylation (H3K27me2) loss in the differential diagnosis of MPNST [12]. Marchione et al. investigated H3K27me2 IHC in MPNST, K27M-mutant glioma, ependymoma, and Merkel cell carcinoma, which were characterized by the loss of H3K27me3. They demonstrated that while H3K27me3 loss is common across these tumor types, H3K27me2 loss is limited to MPNST. Moreover, they also investigated H3K27me2 IHC in cases of malignant melanoma and SS, which were histologic mimics of MPNST and showed various degrees of H3K27me3 loss. As a result, while global H3K27me3 loss was not observed in these tumors, weak and limited H3K27me3 staining was common. On the other hand, H3K27me2 staining was more clearly retained in all cases. Thus, H3K27me2 may be a useful marker for the differential diagnosis of MPNST instead of H3K27me3, the partial loss of which is particularly difficult to assess using immunoreactivity.

Indeed, the present study revealed that some non-MPNSTs showed H3K27me3 partial loss ranging from 10% to 90% of tumor cells. Among them, MPNSTs tended to exhibit *NF1* and/or *p16* deletion more frequently than non-MPNSTs. In particular, the combination of H3K27me3 IHC and *NF1*/*p16* deletion FISH was able to reach an accurate diagnosis in approximately 90% of MPNST cases.

Nevertheless, there were some non-MPNST cases that were difficult to differentiate from MPNST according to the status of H3K27me3 loss and *NF1* and/or *p16* deletion, but these cases can be diagnosed by combining other IHC and FISH findings. For example, SS-3 and SS-9 cases showed *NF1* deletion, and SS-9 cases also exhibited complete loss of H3K27me3. SS is especially important for differential diagnosis from MPNST because of the monomorphic appearance of spindle cell proliferation. However, we were able to reliably distinguish these two cases from MPNST by cytokeratin expression and identifying the *SS18-SSX* fusion gene. In another example, some LMSs (LMS-1, 3, 5) also showed *NF1* and/or *p16* deletion, although positivity for myogenic markers on IHC helped us to reach a correct diagnosis. We often find that the differential diagnosis of MFS and UPS is the most challenging because these tumors have no specific markers on IHC. A recent study revealed that somatic tumor genomic profiles by FoundationOne® CDx showed a high prevalence of *p16/CDKN2A* alterations in MPNST, MFS, and UPS [13]. These sarcomas may have some overlap of genetic features. From the clinical point of view, MFSs are relatively distinguishable according to their superficial localization and characteristic radiographic findings showing extension along the fascia. Ultimately, it seems that there are certain UPS cases that are tremendously difficult to distinguish from MPNST. We re-evaluated the MFS and UPS cases showing co-deletion of *NF1* and *p16* (MFS-5, 10, UPS-2, 5, 6) and confirmed that they were morphologically typical MFS and UPS and different from MPNST when judged comprehensively. In such a situation, we need a multidisciplinary approach that combines not only pathological findings but also epidemiological, clinical, and radiographic findings for an accurate diagnosis.

It has been reported that loss of p16 expression on IHC is associated with poor prognosis in patients with high-grade sarcoma including MPNST [6, 14]. In addition, *p16* homozygous deletion is a marker of poor prognosis in patients with Ewing sarcoma [15]. Moreover, *p16* deletion has been revealed to be a biomarker for poor prognosis in patients with soft tissue sarcoma [13]. In this way, *p16* is an important marker of the clinical behavior of patients with soft tissue sarcoma. In this study, we revealed that the presence of *NF1* and/or *p16* deletion was associated with the histological grade of MPNST. Although we did not investigate the association between *p16* deletion and prognosis directly, *p16* deletion might be an important biological factor associated with the histological grade of MPNST that can be used to predict prognosis.

## Conclusion

FISH for *NF1* and *p16* deletions, which are observed frequently in high-grade MPNSTs, might be a useful ancillary diagnostic tool for differentiating MPNST from other mimicking spindle cell and pleomorphic sarcomas.

## Data Availability

Not applicable.

## Abbreviations

FISH: Fluorescence *in situ* hybridization
H3K27me3: Histone 3 trimethylated on lysine 27
IHC: Immunohistochemistry
LMS: Leiomyosarcoma
MFS: Myxofibrosarcoma
MPNST: Malignant peripheral nerve sheath tumor
NF1: Neurofibromatosis 1
NF1M: Neurofibromatosis 1-associated malignant peripheral nerve sheath tumor
NNF1M: Non-neurofibromatosis 1-associated malignant peripheral nerve sheath tumor
SS: Synovial sarcoma
UPS: Undifferentiated pleomorphic sarcoma
